# Endocrinology in the time of COVID-19: Management of pituitary tumoursThis manuscript is part of a commissioned series of urgent clinical guidance documents on the management of endocrine conditions in the time of COVID-19. This clinical guidance document underwent expedited open peer review by Cesar Boguszewski (*Federal University of Parana, Curitiba,* Brazil), Jens-Otto Jorgensen (Aarhus University Hospital, Denmark), Dominique Maiter (Université catholique de Louvain and Cliniques Universitaires Saint-Luc, Brussels, Belgium), and Ken Ho (*University of New South Wales and St. Vincent's Hospital, Sydney,* Australia)

**DOI:** 10.1530/EJE-20-0473

**Published:** 2020-07-01

**Authors:** Maria Fleseriu, Olaf M Dekkers, Niki Karavitaki

**Affiliations:** 1 Departments of Medicine (Endocrinology) and Neurological Surgery and Pituitary Center, Oregon Health & Science University, Portland, Oregon, USA; 2 Department of Clinical Epidemiology, Leiden University Medical Centre, Leiden, The Netherlands; 3 Department of Endocrinology, Leiden University Medical Centre, Leiden, The Netherlands; 4 Department of Clinical Epidemiology, Aarhus University Hospital, Aarhus, Denmark; 5 Institute of Metabolism and Systems Research, College of Medical and Dental Sciences, University of Birmingham, Birmingham, UK; 6 Centre for Endocrinology, Diabetes and Metabolism, Birmingham Health Partners, Birmingham, UK; 7 Department of Endocrinology, Queen Elizabeth Hospital, University Hospitals Birmingham NHS Foundation Trust, Birmingham, UK

## Abstract

Patients with pituitary tumours, ensuing hormonal abnormalities and mass effects are usually followed in multidisciplinary pituitary clinics and can represent a management challenge even during the times of non-pandemic. The COVID-19 pandemic has put on hold routine medical care for hundreds of millions of patients around the globe, while many pituitary patients' evaluations cannot be delayed for too long. Furthermore, the majority of patients with pituitary tumours have co-morbidities potentially impacting the course and management of COVID-19 (e.g. hypopituitarism, diabetes mellitus, hypertension, obesity and cardiovascular disease). Here, we summarize some of the diagnostic and management dilemmas encountered, and provide guidance on safe and as effective as possible delivery of care in the COVID-19 era. We also attempt to address how pituitary services should be remodelled in the event of similar crises, while maintaining or even improving patient outcomes. Regular review of these recommendations and further adjustments are needed, depending on the evolution of the COVID-19 pandemic status. We consider that the utilization of successful models of pituitary multidisciplinary care implemented during the COVID-19 pandemic should continue after the crisis is over by using the valuable and exceptional experience gained during these challenging times.

## Introduction

Management of patients with pituitary tumours and their associated hormonal abnormalities and mass effect complications can be challenging even during normal times and needs to be preferably delivered in Pituitary Centres of Excellence, especially in complex cases (1). Here, we review some of the diagnostic and management dilemmas that might be encountered in patients with pituitary tumours in the COVID-19 era and we provide guidance on effective and safest delivery of their care. We emphasize that regular review of these recommendations and further adjustments are needed depending on the evolution of the COVID-19 pandemic status.

## What is the expected impact of COVID-19 on patients with pituitary tumours?

- There is currently no proven concern that pituitary tumours *per se* affect the immune system, apart from corticotroph adenomas causing cortisol excess. Patients with uncontrolled Cushing's disease are at higher risk of infections, which affects their mortality risk (2).
- A number of patients with pituitary tumours have co-morbidities potentially impacting the course and management of COVID-19 infections (e.g. hypopituitarism, diabetes mellitus, hypertension, obesity and cardiovascular diseases) (3). Patients with adrenal insufficiency infected with COVID-19 require special attention/care and appropriate steroid cover. For further guidance see (4).
- On the other hand, some patients may have concerns on the safety of face-to-face contact with medical staff and other healthcare professionals, particularly in the hospital setting. This anxiety may delay diagnostic procedures and early management of pituitary tumours with potentially significant adverse sequelae.

## How to manage patients with pituitary tumours without easy access to full investigations in the COVID-19 era

### 1 Acutely unwell patients

**Adrenal crisis:** This scenario is extensively discussed elsewhere (4). It should be pointed out that administration of stress-dose steroids should never be delayed in patients with suspected adrenal crisis.

#### Pituitary apoplexy

- Rely on typical clinical picture for initial assessment if availability of full investigations is limited; manifestations of pituitary apoplexy can include any of the following: acute-onset severe headache, nausea/vomiting, visual loss, diplopia, ptosis and impaired consciousness (5).
- Clinical evaluation of visual fields/acuity, cranial nerves and level of consciousness will help to further substantiate the diagnosis of pituitary apoplexy and its severity.
- Supportive measures to ensure haemodynamic stability and stress dose of glucocorticoids should be offered (5).
- Arrange urgent brain CT to facilitate differential diagnosis (particularly from subarachnoid haemorrhage and meningitis) and pituitary MRI, if further imaging evaluation is deemed necessary (6). Preoperative pituitary MRI is not mandatory if urgent decompression is required.
- Continuous, careful neurological and neuro-ophthalmological monitoring along with regular review of blood electrolytes are needed.
- In cases with mild visual dysfunction, a conservative approach with high-dose glucocorticoids, using their anti-inflammatory and anti-oedematous effects, could be considered (5), especially if access to surgical care is limited. However, very close monitoring is required, and if visual function does not improve quickly or deteriorates, surgical decompression should be performed (after testing for COVID-19 infection).

**Electrolyte disturbances** due to alterations in fluid balance in patients with diabetes insipidus. This scenario is extensively discussed elsewhere (7).

### 2 Patients with newly diagnosed tumours

In all cases, we emphasize the need for history taking and clinical assessment (in a virtual visit, aiming to identify presence of co-morbidities), especially when full biochemical or radiological assessment may not be readily available. A recommended assessment of basic pituitary hormone profile (8) includes: morning blood cortisol, TSH, fT4, prolactin, IGF-I and total testosterone in men and, if clinical suspicion, screening tests for Cushing's disease.

**Tumours with mass effects and/or hormonal hypersecretion:** Newly diagnosed pituitary tumours may present with mass effects (mainly visual deterioration, headaches and hypopituitarism) and/or hormonal hypersecretion (8). During the diagnostic approach, patient face-to-face visits need to be minimized as much as possible and, if needed, should take place with the appropriate protection measures and procedures (aiming to reduce the risk of COVID-19 transmission to patients and hospital staff).

#### Diagnostic approach

- Hypopituitarism: Assessment of ACTH and TSH function (by measuring morning cortisol, TSH and fT4 – a short Synacthen (Cortrosyn) test could be considered in cases of equivocal morning cortisol levels if less risks of COVID-19 in that particular centre) and replacement therapy if needed (as per Endocrine Society guidelines (9)).
- Visual dysfunction: Clinical evaluation of visual fields/acuity and of cranial nerves and formal assessment of visual fields.
- Measurement of prolactin and IGF-I; screening tests for Cushing's disease only if clinically indicated.
- Head CT or pituitary MRI with contrast (the latter only if urgent and can be done safely).

#### Management

- In all types of pituitary tumours (except prolactinomas) causing severe visual deterioration, surgery is the treatment of choice (10). However, prior to surgical intervention, assessment of COVID-19 status is needed. In countries where viral load is low, the decision on assessment of COVID-19 status should be taken by the relevant multidisciplinary team and rely on national guidance.
- Patients with mild acromegaly (as indicated by clinical picture and mild/moderately elevated IGF-I levels) and tumour, not causing visual deterioration, can wait for further evaluation/management at a later stage. A virtual visit should be sufficient for clinical evaluation and discussion of the condition, co-morbidities and possibly alleviate patient's stress if they are made aware of the potential complications of GH excess.
- Patients with severe acromegaly (as indicated by clinical picture and significantly elevated IGF-I levels) attributed to a tumour not causing visual deterioration are candidates for medical treatment. Results of investigations, treatment plans and management targets can be discussed in a virtual clinic setting. Medical treatment can include short- or long-acting somatostatin receptor ligands (SRLs) (octreotide 100–200 mcg tds s.c. which is easy to self-inject, lanreotide 120 mg deep s.c. injection every 6–8 weeks, which may also be self-injected, octreotide LAR 30 mg every 6–8 weeks i.m. and growth hormone receptor antagonist, pegvisomant, in function of country availability and regulatory approval) (6). We recommend an individualized approach to treatment; however, starting with higher SRLs doses aiming to reduce the frequency of injections (and thus contact with healthcare professionals) should be attempted. Pegvisomant s.c. with gradual dose titration can be used in the case of small tumours and normal liver function. Addition of cabergoline could be considered if there is no response to the previously mentioned treatments. In some countries, depending on regulatory approval, cabergoline could also be used as first-line treatment (in cases of mild acromegaly or prolactin co-secreting tumours). Overall, dose titration during the COVID-19 pandemic should mainly rely on clinical status, IGF-I measurement (when safe to arrange) and adverse effects (Table 1). Training of the patients or family members on administration of injections could be offered by online visits or by video, and if not possible, injections could be administered by a nurse in a clinic (provided the patient does not belong to the vulnerable group of people requiring strict adherence to social isolation rules).
- For patients with acromegaly not having achieved biochemical control before the end of the pandemic, a decision should be made to balance pros and cons of changing dosing/interval regimen in the absence of face-to-face visits and further laboratory evaluations. While dose adjustment is usually based on IGF-I, in high-risk patients, use of virtual visit clinical evaluation and review of relevant symptoms/signs should be attempted.
- Cushing's disease (for further guidance see (11)).
- In all cases of Cushing's disease and acromegaly, intensive treatment of co-morbidities (e.g. hypertension, diabetes mellitus and heart failure) is strongly recommended.
- Macroprolactinomas causing visual deterioration should be treated with dopamine agonists; dose titration and monitoring of treatment should rely on tolerability of these agents (including watching for manifestations of impulse control disorders and worsening depression) and improvement of visual dysfunction (with self-reported improvement by the patient in case of virtual visits or by formal visual field assessment 2–3 weeks after commencing treatment if clinically indicated).
- Management of symptomatic microprolactinomas or macroprolactinomas not causing visual dysfunction may be postponed for 6 months in most patients. However, low-dose dopamine agonist can be initiated by virtual visit, for example, in a woman with recently diagnosed microprolactinoma causing infertility who is trying to become pregnant for a long period and has no contraindications for this treatment.

**Table 1:** Clinical evaluation of medical treatment for prolactinomas and acromegaly in the absence of biochemical monitoring.

	General considerations	Signs of uncontrolled hormone excess	Side effects
Prolactinomas			
*Dopamine agonists*	Evaluation for impulse control disorders and worsening depression	Women: galactorrhoea and irregular periods Males: manifestations of testosterone deficiency	Nausea, dizziness and constipation
Acromegaly			
*Somatostatin receptor ligands*	Unless patients are self-injecting, the interval between injections can be prolonged and doses slightly increased as needed during the COVID-19 pandemic	Increased sweating, headaches and tiredness	Nausea, diarrhoea and abdominal pain
*Pegvisomant*	Doses can be easily adjusted as self-administered	Increased sweating, headaches and tiredness	Reaction to injection sites and abnormal liver function tests

**Tumours without mass effects and without hormonal hypersecretion:** Management of these tumours can be deferred for several months (ideally less than 6 months).

## Patients who require regular/routine monitoring

- Working in a multidisciplinary fashion (including, but not limited to neurosurgery, neuro-endocrinology and neuro-ophthalmology) is essential to stratify patients' risk, guide intensity of monitoring and ensure delivery of timely care to those in need of laboratory investigations or surgery.
- Furthermore, close collaboration with the health system's managerial/administration team is vital for securing appropriate resources, prioritization of appointments and for smooth implementation of alternative means of delivery of care (e.g. virtual clinics).
- In patients with functioning pituitary tumours in remission or well controlled on medical treatment, follow-up with virtual clinic appointments is recommended. Generally, treatment regimens should not be changed for a period of 6 months, unless there is a strong clinical suspicion of significant changes in the response to therapy or presence of adverse effects (Table 1). A potential exception could be patients with acromegaly controlled on long-acting SRLs. In this group, an increase in their dose aiming to reduce the frequency of injections should be considered, as rates of adverse events seem to be similar.
- Plans for radiotherapy during the COVID-19 pandemic need to be postponed for 6 months with review of further plans later.
- Frequency of image monitoring needs to be minimized to reduce the risk of COVID-19 transmission to patients and hospital staff, as well as to reduce the workload of the Neuroradiology Department. Follow-up imaging of tumours not touching the optic pathway could be deferred for 6 months. For tumours abutting or compressing the optic chiasm, clinical monitoring of vision (including self-reported information by the patient or formal assessment of visual fields) is recommended. Imaging for functioning pituitary tumours, well-controlled by medical treatment, is not advised as hormonal and tumour mass responses are only rarely discordant.
- In patients with pituitary tumours diagnosed with COVID-19 infection, an urgent virtual clinic appointment is recommended, aiming to cover the implications of COVID-19 infection in the setting of cortisol deficiency, presence of various co-morbidities (e.g. obesity, hypertension, diabetes mellitus and cardiovascular diseases) and presence of adverse effects of medical treatments (e.g. gastrointestinal side effects, liver dysfunction related with medical treatment for acromegaly). In the last scenario, stopping or postponing the administration of the responsible drug is recommended.
- Dose adjustments of pituitary hormone replacement could be performed by clinical assessment rather than laboratory investigations for a period of 6 months in most cases (Table 2). If supplies are not available, discontinuation of GH and gonadal hormone replacement for a short period should not affect long-term outcomes. In such cases, the patients need to be informed about symptoms/signs that they may possibly experience and also be reassured that these do not pose risks to their health.

**Table 2:** Clinical evaluation of hormone replacement therapy in the absence of biochemical monitoring (for steroid substitution see AI paper, for desmopressin see DI paper).

	General considerations	Signs of overdosing	Signs of underdosing
Thyroid hormone substitution replacement	Levothyroxine: Long half-life (~7 days)	Tachycardia, tremor, weight loss, anxiety, diarrhoea and insomnia	Weight gain, dry skin, constipation, lethargy and fatigue
GH replacement	Short term discontinuation does not affect long-term outcomes	Headaches, carpal tunnel syndrome, sweating and oedema	Tiredness
Estrogen replacement in women	Gonadal hormone replacement could be stopped for a short period and patients need to be informed of the symptoms/signs they may experience but also be reassured that these do not pose risks to their health	N/A	Hot flushes
Testosterone replacement in men	Treatment could be stopped for a short period if follow-up for optimal and safe replacement is not possible in elderly patients	Symptoms of prostatic enlargement (e.g. nocturia) and manifestations of polycythaemia	Fatigue and mood changes

## Transsphenoidal surgery in the COVID-19 pandemic era

- Based on very recent, but still anecdotal data, endonasal surgery (endoscopic or microscopic) for COVID-19 positive patients with pituitary tumours is considered a high-risk procedure (12). As a result of this, several neurosurgical groups across the world are currently undertaking only urgent surgeries and postponing elective surgeries.
- Testing for COVID-19 infection is strongly recommended 48 h prior to transsphenoidal surgery. If the results are positive, deferring surgery until infection is cleared needs to be considered. If this is not possible, appropriate personal protective equipment (PPE) for anyone in the operating theatre is recommended (12). Furthermore, given the possibility of false-negative results for COVID-19 testing, the surgical theatre team should still wear full PPE even in COVID-19 negative cases, as these surgical procedures are aerosol generating.

## How should current patients with pituitary tumours be advised about risk?

- Patients with Cushing's disease should be informed of the impact of cortisol excess on their immune response and the risk of contracting COVID-19 infection. Strict adherence to social isolation rules as defined by each country needs to be emphasized to this group (11).
- Patients with cortisol deficiency should be clearly informed of the implications of their condition should they become COVID-19 positive and optimal management plans. Strict adherence to social isolation rules needs to be emphasized to this group (4).
- Patients with acromegaly or Cushing's may have co-morbidities like hypertension, obesity and diabetes mellitus and should be informed of the impact of them on the prognosis of COVID-19 infection. The importance of optimal hormonal control with medical treatment and of the management of each co-morbidity should be highlighted in the virtual discussions with patients. General recommendations for social distancing should also apply for these patients.
- Patients with diabetes insipidus need to be informed of the risk of disturbances in the electrolyte and fluid balance in the context of COVID-19 infection (7).

## How should pituitary services for the condition be remodelled in the acute crisis?

- Routine care of patients with pituitary adenomas can be offered remotely (telephone or video clinics) avoiding face-toface contact and the risks associated with this in the COVID-19 era. Face-to-face visits could be postponed in most cases for 3–6 months without compromising optimal care. Through this model of care, patients should also be informed about situations requiring immediate contact with the endocrine service (e.g. manifestations of pituitary apoplexy, of tumour growth and of suboptimal pituitary hormonal replacement), risks related with COVID-19 infection and relevant measures each government and healthcare system has applied during the pandemic (e.g. social isolation).
- An endocrine help-line run by endocrinologists and endocrine nurses should be available 24/7 aiming to offer immediate support to patients with urgent problems/queries, to reduce anxiety and to reduce workload of primary care clinicians.
- Further triage of patients who might need closer follow-up and/or laboratory work-up can be done also by telemedicine appointments. Laboratory investigations, if deemed relevant for clinical decisions, can take place close to home (or in some countries, blood sampling can be exceptionally performed at home for at-high-risk patients) and transferred to the specialist centre.
- Dissemination of information via patient support groups' websites is strongly recommended.

## What might be the longer-term consequence for service provision?

- As the backlog of patients to be evaluated face to face and to have surgery will be significant, a second triage period after 3 months is needed. This is also important as non-urgent- or semi-urgent issues could become urgent. Patients should also be encouraged to keep a diary of their symptoms and call providers if any changes occur over time.
- Shortage of medication availability may be an issue for specific drugs in the future and clinicians need to be alert and proactive if such problems are suspected to appear during the COVID-19 era. Particularly, if such circumstances arise, GH and gonadal hormone replacement could be stopped for a short period and patients need to be informed of the symptoms/signs they may experience, but also be reassured that these do not pose risks to their health in the short term.
- If, in the immediate future, issues with testing patients and getting results for COVID-19 within 24 h, as well as protective equipment shortage are all resolved, this could change the optimal management balance towards carefully considering patients who are good candidates for surgery, especially in acromegaly, Cushing's disease and some prolactinomas.
- Revision of the pituitary centres and pituitary line services provisions and utilization of successful models of multidisciplinary care implemented during the COVID-19 pandemic are recommended at a later stage, aiming to exploit the valuable experience gained during these challenging times.

## Disclaimer

Due to the emerging nature of the COVID-19 crisis, this document is not based on extensive systematic review or meta-analysis but on rapid expert consensus. The document should be considered as guidance only, and it is not intended to determine an absolute standard of medical care. Healthcare staff need to consider individual circumstances when devising the management plan for a specific patient.

## Declaration of interest

Olaf Dekkers is Deputy Editor of the *European Journal of Endocrinology*. He was not involved in the editorial or peer-review process of this paper, on which he is listed as an author. Maria Fleseriu has received research support as principal investigator to Oregon Health & Science University from Chiasma, Crinetics, Ionis, Novartis and as an occasional scientific consultant from Chiasma, Crinetics, Ipsen, Novartis and Pfizer. Niki Karavitaki has received research support and educational grants from Pfizer, Novartis, Shire and Ipsen.

## Funding

This research did not receive any specific grant from any funding agency in the public, commercial or not-for-profit sector.
